# Supplementary material for: Pharmacometabolomics in TB meningitis—Understanding the pharmacokinetic, metabolic, and immune factors associated with anti-TB drug concentrations in cerebrospinal fluid
Source: PLoS One. 2025 Mar 3;20(3):e0315999. doi: 10.1371/journal.pone.0315999 (PMC11875335; doi:10.1371/journal.pone.0315999)
Supplement: S1 Table — (DOCX) [file pone.0315999.s001.docx]

| **Supplementary Table 1: Serum and CSF drug concentrations 2 and 6 hours after most recent antibiotic dose** | | | |
| --- | --- | --- | --- |
|  |  |  |  |
| Antibiotic | | Serum concentration (mg/L)  Median (range) | CSF concentration (mg/L)  Median (range) |
| Cycloserine | |  |  |
|  | 2 hours | 36.6 (19-98.6) | 16.1 (10.4-46.3) |
|  | 6 hours | 31.4 (15.7-60.5) | 18.2 (11.3-33.8) |
| Ethambutol | |  |  |
|  | 2 hours | 1.5 (1.0-2.3) | 0.09 (0.03-0.15) |
|  | 6 hours | 1.7 (1.2-3.1) | 0.16 (0.06-0.3) |
| Imipenem | |  |  |
|  | 2 hours | 5.9 (2.7-19.4) | 0.8 (0.03-3.8) |
|  | 6 hours | 3.4 (2.3-16.6) | 0.5 (0.02-2.7) |
| Isoniazid | |  |  |
|  | 2 hours | 1.5 (0.3-3.9) | 0.5 (0.04-2.5) |
|  | 6 hours | 1.6 (0.5-3.6) | 0.9 (0.5-3.1) |
| Levofloxacin | |  |  |
|  | 2 hours | 7.7 (1.2-14.1) | 1.4 (0.4-7.3) |
|  | 6 hours | 7.7 (2.8-15.8) | 3.4 (1.2-11.6) |
| Linezolid | |  |  |
|  | 2 hours | 6.7 (1.5-15.1) | 0.7 (0.1-3.6) |
|  | 6 hours | 6.8 (2.3-12.0) | 2.9 (0.7-5.3) |
| Moxifloxacin | |  |  |
|  | 2 hours | 2.7 (1.6-4.1) | 0.8 (0.2-1.5) |
|  | 6 hours | 3.8 (1.3-4.9) | 1.0 (0.7-1.6) |
| Pyrazinamide | |  |  |
|  | 2 hours | 29.1 (17.9-69.5) | 21.7 (9.6-49.9) |
|  | 6 hours | 33.3 (15.6-56.7) | 29.2 (20.2-54.9) |
| Rifampin | |  |  |
|  | 2 hours | 3.2 (1.6-7.0) | 0.06 (0.02-0.3) |
|  | 6 hours | 4.1 (0.9-9.3) | 0.19 (0.05-0.41) |
