# Supplementary material for: Pharmacometabolomics in TB meningitis—Understanding the pharmacokinetic, metabolic, and immune factors associated with anti-TB drug concentrations in cerebrospinal fluid
Source: PLoS One. 2025 Mar 3;20(3):e0315999. doi: 10.1371/journal.pone.0315999 (PMC11875335; doi:10.1371/journal.pone.0315999)
Supplement: S2 Table — (DOCX) [file pone.0315999.s002.docx]

| **Supplementary Table 2: Association between clinical and demographic factors and serum drug concentrations** | | | | | |
| --- | --- | --- | --- | --- | --- |
|  |  |  |  |  |  |
|  |  | Univariate Analysis | | Multivariate Analysis | |
| Antibiotic | | Parameter Estimate (standard error) | p-value | Parameter Estimate (standard error) | p-value |
| Cycloserine | |  |  |  |  |
|  | Age | -2.5 (0.5) | 0.002 | 0.8 (1.8) | 0.65 |
|  | Male Sex | 51.1 (8.5) | <0.001 | 65.9 (33.8) | 0.05 |
|  | Weight | 0.7 (0.8) | 0.39 |  |  |
|  | Creatinine | 0.4 (0.7) | 0.56 |  |  |
|  | 6-hour Sample time point | -8.8 (7.3) | 0.25 |  |  |
| Ethambutol | |  |  |  |  |
|  | Age | 0.03 (0.02) | 0.14 |  |  |
|  | Male Sex | -0.4 (0.4) | 0.37 |  |  |
|  | Weight | 0.02 (0.04) | 0.57 |  |  |
|  | Creatinine | -0.01 (0.01) | 0.35 |  |  |
|  | 6-hour Sample time point | 0.2 (0.3) | 0.42 |  |  |
| Imipenem | |  |  |  |  |
|  | Age | -0.08 (0.07) | 0.27 |  |  |
|  | Male Sex | -0.2 (1.7) | 0.92 |  |  |
|  | Weight | -0.08 (0.08) | 0.35 |  |  |
|  | Creatinine | -0.02 (0.08) | 0.86 |  |  |
|  | 6-hour Sample time point | -3.3 (1.4) | 0.02 |  |  |
| Isoniazid | |  |  |  |  |
|  | Age | -0.02 (0.02) | 0.3 |  |  |
|  | Male Sex | 0.07 (0.4) | 0.85 |  |  |
|  | Weight | 0.03 (0.02) | 0.18 |  |  |
|  | Creatinine | -0.01 (0.01) | 0.3 |  |  |
|  | 6-hour Sample time point | 0.2 (0.2) | 0.3 |  |  |
| Levofloxacin | |  |  |  |  |
|  | Age | 0.02 (0.05) | 0.7 |  |  |
|  | Male Sex | 0.4 (1.2) | 0.73 |  |  |
|  | Weight | 0.07 (0.06) | 0.3 |  |  |
|  | Creatinine | 0.02 (0.04) | 0.6 |  |  |
|  | 6-hour Sample time point | -0.03 (0.8) | 0.97 |  |  |
| Linezolid | |  |  |  |  |
|  | Age | -0.1 (0.05) | 0.007 |  |  |
|  | Male Sex | -0.9 (1.3) | 0.53 |  |  |
|  | Weight | 0.04 (0.06) | 0.54 |  |  |
|  | Creatinine | 0.02 (0.04) | 0.79 |  |  |
|  | 6-hour Sample time point | -0.004 (0.9) | 1 |  |  |
| Moxifloxacin | |  |  |  |  |
|  | Age | -0.002 (0.03) | 0.93 |  |  |
|  | Male Sex | 0.7 (0.32) | 0.34 |  |  |
|  | Weight | -0.004 (0.05) | 0.94 |  |  |
|  | Creatinine | -0.001 (0.008) | 0.9 |  |  |
|  | 6-hour Sample time point | 0.01 (0.2) | 0.93 |  |  |
| Pyrazinamide | |  |  |  |  |
|  | Age | -0.3 (0.2) | 0.1 | -0.1 (0.2) | 0.64 |
|  | Male Sex | -4.1 (5.3) | 0.44 |  |  |
|  | Weight | 0.07 (0.3) | 0.81 |  |  |
|  | Creatinine | -0.4 (0.2) | 0.08 | -0.3 (0.2) | 0.15 |
|  | 6-hour Sample time point | -1.1 (3.1) | 0.73 |  |  |
| Rifampin (median, range) | |  |  |  |  |
|  | Age | -0.03 (0.02) | 0.27 |  |  |
|  | Male Sex | -0.6 (0.6) | 0.37 |  |  |
|  | Weight | 0.05 (0.04) | 0.28 |  |  |
|  | Creatinine | -0.03 (0.02) | 0.31 |  |  |
|  | 6-hour Sample time point | 0.7 (0.5) | 0.19 |  |  |
