# Supplementary material for: Pharmacometabolomics in TB meningitis—Understanding the pharmacokinetic, metabolic, and immune factors associated with anti-TB drug concentrations in cerebrospinal fluid
Source: PLoS One. 2025 Mar 3;20(3):e0315999. doi: 10.1371/journal.pone.0315999 (PMC11875335; doi:10.1371/journal.pone.0315999)
Supplement: S3 Table — (DOCX) [file pone.0315999.s003.docx]

| **Supplementary Table 3: Association between clinical and demographic factors and CSF drug concentrations** | | | | | |
| --- | --- | --- | --- | --- | --- |
|  |  |  |  |  |  |
|  |  | Univariate Analysis | | Multivariate Analysis | |
| Antibiotic | | Parameter Estimate  (standard error) | p-value | Parameter Estimate  (standard error) | p-value |
| Cycloserine | |  |  |  |  |
|  | Age | -1.2 (0.2) | 0.003 | -0.02 (0.6) | 0.98 |
|  | Male Sex | 24 (3.4) | 0.001 | 10.7 (13.2) | 0.42 |
|  | Weight | 0.1 (0.4) | 0.71 |  |  |
|  | Creatinine | 0.4 (0.2) | 0.17 |  |  |
|  | 6-hour Sample time point | -0.02 (2.6) | 0.99 |  |  |
|  | Serum drug concentration | 0.4 (0.05) | <0.001 | 0.25 (0.07) | <0.001 |
| Ethambutol | |  |  |  |  |
|  | Age | 0.009 (0.002) | 0.005 | 0.007 (0.002) | <0.001 |
|  | Male Sex | 0.1 (0.06) | 0.12 | 0.04 (0.03) | 0.18 |
|  | Weight | 0.003 (0.005) | 0.57 |  |  |
|  | Creatinine | 0.001 (0.002) | 0.59 |  |  |
|  | 6-hour Sample time point | 0.07 (0.02) | 0.007 | 0.08 (0.02) | <0.001 |
|  | Serum drug concentration | 0.03 (0.03) | 0.29 |  |  |
| Imipenem | |  |  |  |  |
|  | Age | 0.007 (0.02) | 0.69 |  |  |
|  | Male Sex | 0.8 (0.3) | 0.03 |  |  |
|  | Weight | -0.02 (0.02) | 0.35 |  |  |
|  | Creatinine | 0 (0.02) | 0.87 |  |  |
|  | 6-hour Sample time point | -0.4 (0.3) | 0.13 |  |  |
|  | Serum drug concentration | 0.01 (0.03) | 0.65 |  |  |
| Isoniazid | |  |  |  |  |
|  | Age | -0.003 (0.01) | 0.81 |  |  |
|  | Male Sex | 0.2 (0.3) | 0.57 |  |  |
|  | Weight | 0.03 (0.02) | 0.16 |  |  |
|  | Creatinine | -0.002 (0.01) | 0.77 |  |  |
|  | 6-hour Sample time point | 0.6 (0.2) | 0.001 | 0.6 (0.08) | p<0.001 |
|  | Serum drug concentration | 0.7 (0.09) | <0.001 | 0.5 (0.1) | p<0.001 |
| Levofloxacin | |  |  |  |  |
|  | Age | 0.05 (0.04) | 0.2 |  |  |
|  | Male Sex | 2.2 (0.8) | 0.01 | 2 (0.7) | 0.004 |
|  | Weight | 0.04 (0.05) | 0.41 |  |  |
|  | Creatinine | 0.03 (0.02) | 0.3 |  |  |
|  | 6-hour Sample time point | 1.7 (0.3) | <0.001 | 1.7 (0.3) | <0.001 |
|  | Serum drug concentration | 0.3 (0.1) | 0.002 | 0.2 (0.06) | <0.001 |
| Linezolid | |  |  |  |  |
|  | Age | -0.04 (0.02) | 0.09 | 0.01 (0.02) | 0.48 |
|  | Male Sex | -0.2 (0.5) | 0.75 |  |  |
|  | Weight | 0.02 (0.02) | 0.53 |  |  |
|  | Creatinine | 0.003 (0.03) | 0.66 |  |  |
|  | 6-hour Sample time point | 1.7 (0.3) | <0.001 | 1.8 (0.2) | <0.001 |
|  | Serum drug concentration | 0.3 (0.06) | <0.001 | 0.3 (0.04) | <0.001 |
| Moxifloxacin | |  |  |  |  |
|  | Age | -0.01 (0.008) | 0.14 | -0.01 (0.008) | 0.18 |
|  | Male Sex | 0.4 (0.2) | 0.06 |  |  |
|  | Weight | 0.02 (0.02) | 0.4 |  |  |
|  | Creatinine | 0.02 (0.01) | 0.05 | 0.02 (0.01) | 0.19 |
|  | 6-hour Sample time point | 0.3 (0.1) | 0.1 | 0.1 (0.09) | 0.18 |
|  | Serum drug concentration | 0.2 (0.08) | 0.06 | 0.1 (07) | 0.12 |
| Pyrazinamide | |  |  |  |  |
|  | Age | -0.09 (0.2) | 0.57 |  |  |
|  | Male Sex | 0.2 (3.9) | 0.97 |  |  |
|  | Weight | 0.2 (0.2) | 0.34 |  |  |
|  | Creatinine | -0.1 (0.2) | 0.78 |  |  |
|  | 6-hour Sample time point | 7.6 (3) | 0.02 | 8 (2.3) | 0.001 |
|  | Serum drug concentration | 0.5 (0.1) | <0.001 | 0.5 (0.1) | <0.001 |
| Rifampin (median, range) | |  |  |  |  |
|  | Age | 0.001 (0.002) | 0.56 |  |  |
|  | Male Sex | 0.06 (0.04) | 0.12 |  |  |
|  | Weight | 0.002 (0.002) | 0.38 |  |  |
|  | Creatinine | 0.002 (0.001) | 0.16 |  |  |
|  | 6-hour Sample time point | 0.1 (0.02) | <0.001 | 0.1 (0.02) | <0.001 |
|  | Serum drug concentration | 0.02 (0.006) | 0.003 | 0.02 (0.005) | 0.001 |
